# Radiosensitizing Effect of Schinifoline from *Zanthoxylum schinifolium* Sieb et Zucc on Human Non-Small Cell Lung Cancer A549 Cells: A Preliminary *in Vitro* Investigation

**DOI:** 10.3390/molecules191220128

**Published:** 2014-12-01

**Authors:** Cheng-Fang Wang, Li Fan, Mei Tian, Xue-Song Qi, Jian-Xiang Liu, Jiang-Bin Feng, Shu-Shan Du, Xu Su, Yong-Yan Wang

**Affiliations:** 1 China CDC Key Laboratory of Radiological Protection and Nuclear Emergency, National Institute for Radiological Protection, Chinese Center for Disease Control and Prevention, Beijing 100088, China; E-Mails: wangchengfang@mail.bnu.edu.cn (C.-F.W.); msfl@sina.com (L.F.); tianmei@nirp.cn (M.T.); cedar121@sina.com (X.-S.Q.); jxlamy@gmail.com (J.-X.L.); fengjiangbin@126.com (J.-B.F.); 2 State Key Laboratory of Earth Surface Processes and Resource Ecology, Beijing Normal University, Beijing 100875, China; E-Mails: dushushan@bnu.edu.cn (S.-S.D.); narcissus09@126.com (Y.-Y.W.)

**Keywords:** schinifoline, *Zanthoxylum schinifolium*, radiosensitization, A549, apoptosis, cytofluorimetry

## Abstract

Schinifoline (SF), a 4-quinolinone derivative, was found in *Zanthoxylum schinifolium* for the first time. 4-Quinolinone moieties are thought to have cytotoxic activity and are often used as a tubulin polymerization inhibitors, heterogeneous enzyme inhibitors and antiplatelet agents. However, very little information respect to radiosensitization has focused on SF. This work aimed to investigate the radiosensitizing effect of SF on A549 cells. The cell viability results indicated cytotoxicity of SF on A549 cells, with IC_50_ values of 33.7 ± 2.4, 21.9 ± 1.9 and 16.8 ± 2.2 μg/mL, respectively, after 6, 12, 24 h treatment with different concentrations, and the 10% or 20% IC_50_ concentration during 12 h was applied in later experiments. The results of cell proliferative inhibition and clonogenic assay showed that SF enhanced the radiosensitivity of A549 cells when applied before ^60^Co γ-irradiation and this effect was mainly time and concentration dependent. The flow cytometric data indicated that SF treatment before the irradiation increased the G_2_/M phase, thus improving the radiosensitivity of A549, leading to cell apoptosis. This paper is the first study that describes the *in vitro* radiosensitising, cell cycle and apoptotic-inducing effects of schinifoline.

## 1. Introduction

Lung cancer is a leading cause of cancer deaths in the world, and non-small cell lung cancer (NSCLC) accounts for approximately 85% of all lung cancer cases [1,2,3]. Radiotherapy combined with surgery and chemotherapy is an important treatment and widely used in the clinic against NSCLC. However, it commonly develops resistance to radiation and complications, causing the recurrence and metastasis, leading to treatment failure [4]. Because of the poor overall results, the use of combined-modality treatment is needed to reduce the effects of radioresistance in lung tumors. Herbal medicines may be suited to alleviate the side effects of radiotherapy and chemotherapy and increase the effect of radiation on neoplastic cells [5]. In this context, plants are a potential alternative source of safer radiosensitizer molecules/drugs.

*Zanthoxylum schinifolium* Sieb et Zucc (Rutaceae) is an aromatic plant that is widely used as a pungent condiment and seasoning in China, Japan and other East Asian countries [6]. It is also well known for its medicinal properties, including anticancer activity, anti-platelet aggregation, and anti-inflammatory activity [7,8,9]. Schinifoline (SF), a 4-quinolinone derivative, was isolated from *Z. schinifolium* for the first time [10]. Previous reports have revealed that quinolinone alkaloids possess cytotoxic activity and they are often used as tubulin polymerization inhibitor, heterogeneous enzyme inhibitors and antiplatelet agents [11,12,13]. However, to the best of our knowledge, very little information respect to radiosensitization has focused on SF. Therefore, this work was conducted to evaluate the radiosensitizing effect of SF on human lung adenocarcinoma cells (A549), and the cell cycle and apoptosis were also determined, which could provide the basis for the future mechanism research.

## 2. Results and Discussion

**Figure 1:**
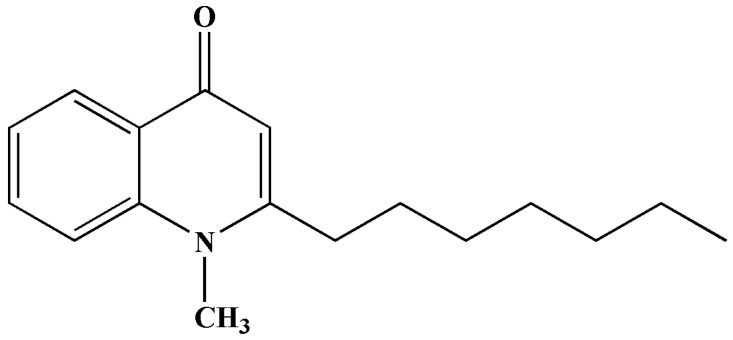
The structure of schinifoline.

**Figure 2:**
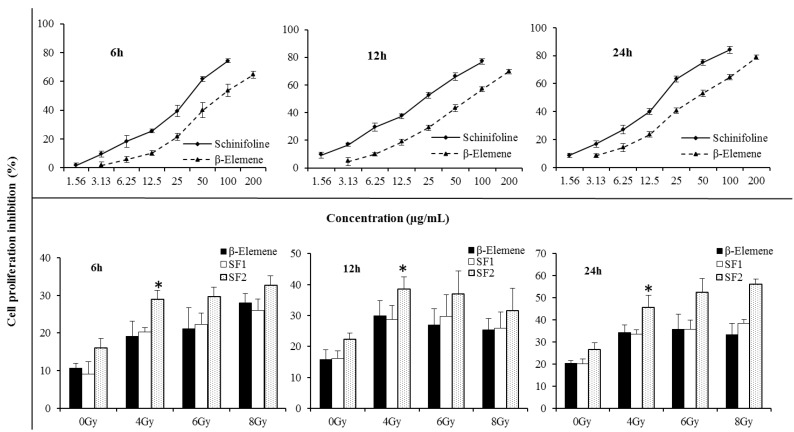
Cell proliferative inhibition A549 by CCK-8 assay. The three curve charts represented cell proliferative inhibition effect of β-elemene/schinifoline ranging from 0 to 200 µg/mL in A549 after 6, 12 and 24 h treatment, respectively. The three bar charts showed cell proliferative inhibition A549 which were treated with β-elemene (15 µg/mL) or schinifoline (SF_1_, 2 µg/mL; SF_2_, 4 µg/mL) alone or in combination with irradiation. The cells were incubated with drugs for 12 h, thereafter, growth media were replaced with fresh medium and the cells were irradiated with single IR doses of 4, 6 and 8 Gy, the cell inhibition was recorded after 6, 12 and 24 h, respectively. Each value is the mean ± SD of six determinations. * *p* < 0.01, compared with SF_1_.

The structure of SF was presented in Figure 1. SF cytotoxicity tests were carried out to optimize the concentration for the radiosensitizing experiments. As shown in Figure 2, SF was found to have a cytotoxic effect on A549 cells. The IC_50_ values were 33.7 ± 2.4, 21.9 ± 1.9 and 16.8 ± 2.2 μg/mL, respectively, after 6, 12, 24 h treatment with different concentrations. β-Elemene (EL) extracted from the traditional Chinese medicine *Curcuma wenyujin* Y.H.Chen et C.Ling (Zingiberaceae) has been developed for injection and emulsion. The injection of EL was used to aid in the treatment of radiotherapy and chemotherapy in clinical and had a synergistic sensitization effect on lung cancer, liver cancer, esophageal cancer, *etc.* Therefore EL was used as the positive control in this paper. In Figure 2 the cell inhibition of SF is stronger than that of EL (positive control), in both a dose- and time-dependent manner. To investigate the radiosensitising effect of SF, cells were incubated with very slightly cytotoxic concentrations, *i.e.*, below the IC_50_ value. According to the results of cytotoxic effect on A549 cells in Figure 2 and previous report of the EL (positive control) on radiosensitization [14,15], concentrations of 10% or 20% IC_50_ during 12 h were applied in the later experiments.

A clear concentration-dependent radiosensitising effect of SF at 4 Gy was observed in A549 cells by CCK-8 assay (Figure 2, Tukey’s tests, *p* < 0.01). The cell proliferative inhibition with SF alone or in combination with IR was higher than EL at 20% IC_50_ value. With the increase of irradiation dose, enhancement of radiosensitization by the test drugs at 12 h was different from 6 h and 24 h. The cell proliferative inhibition during 12 h with treatments by irradiation of 6 or 8 Gy was lower than that exposed to 4 Gy, while during 6 h and 24 h, the cell proliferative inhibition was dose-dependent. Moreover, radiosensitizing effect of drugs in combination with irradiated 8 Gy showed a weak time-dependent effect. Compared with the cell proliferative inhibition of 6 h, a plateau was observed at 12 h, and an increase at 24 h. This might be associated with the direct killing effect of high dose irradiation and cell cycle redistribution. It could be seen that radiosensitizing effect of SF was mainly time and concentration dependent.

**Figure 3:**
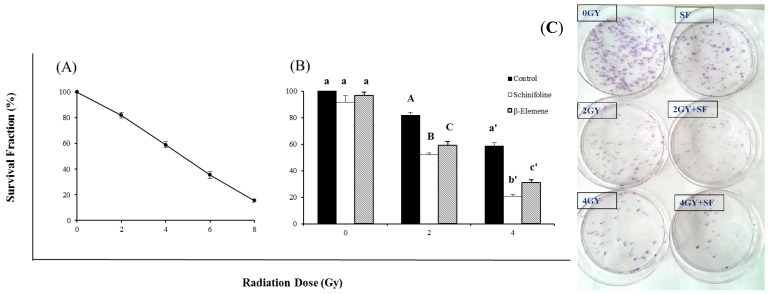
Colony forming assay of A549 cells after exposure to irradiation (IR) or IR in combination with β-elemene/schinifoline. (**A**) The curve represented the cells which were irradiated with different doses of 2, 4, 6 and 8 Gy, respectively. (**B**) Influence of β-elemene (15 µg/mL) and schinifoline (4 µg/mL) on the radiosensitivity of A549 cells. Colony forming efficiency was determined 14 days later and colonies containing at least 50 cells were scored. All data are obtained from three independent experiments, each performed in triplicate. Means under the same exposure dose (0, 2, or 4 Gy) followed by the same letters (a) do not differ significantly or followed by the different letters (A, B, C/a', b', c') differ significantly (*p* < 0.01) in ANOVA and Tukey’s tests. (**C**) The representative picture of colonies.

Figure 3 shows the radiosensitizing efficiency of SF by a clonogenic assay. The colony-forming fraction curve was obtained after exposure to 2, 4, 6 or 8 Gy of γ-radiation. The survival fraction is clearly dose dependent. This radiosensitizing effect was demonstrated by the bar chart that cells were treated with a 20% IC_50_ concentration of SF alone (4 μg/mL) or combined with irradiation (Figure 3). The colony-forming fractions showed that the low concentration of SF had radiosensitizing effect with increasing radiation doses compared to radiation alone and was nearly non-toxic to the cells (Figure 3). Statistical analysis using one-way ANOVA revealed that radiosensitization of SF was significantly stronger than EL (positive control). SF could improve the sensitivity of tumor cells to IR and thus the dose of irradiation could be reduced to half without any decreased inhibitory effect.

**Figure 4:**
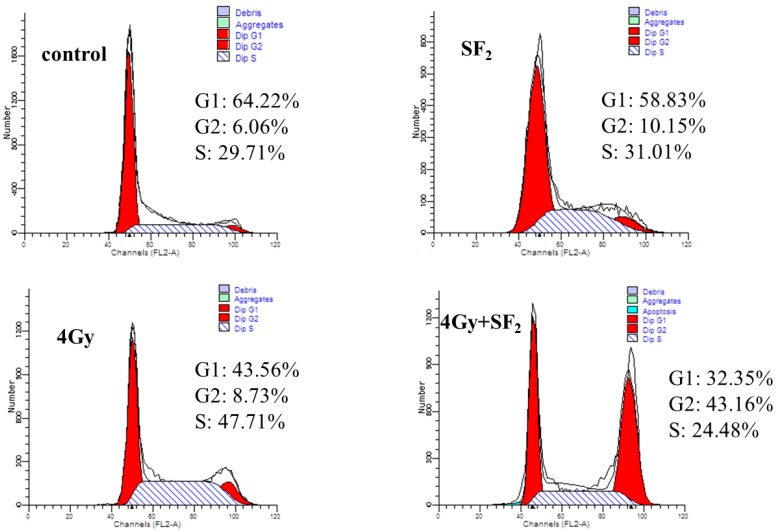
Cell cycle arrest induced by ^60^Co γ-irradiation in A549 cells by the FACS analysis.

It had previously been shown that in the cell cycle, the sensitivity to ionizing radiation varies. Tumor cells usually had been found to be resistant to radiation in the middle to late S phase and in the G_1_ phase, while being sensitive to radiation at mitosis and the G_1_/S-phase border [16]. Therefore, it’s an important way to improve the radiosensitivity of tumor cells by blocking most cells at the mitosis or G_1_/S- phase of the cell cycle. In this work, the effects of SF alone or combined with IR on the cell cycle were accessed and the results are shown in Figure 4 and Table 1. Compared to cells in the control group, SF_2_ (20% IC_50_) treatment for 12 h lowered the percentage of cells in G_1_ and S phase, and increased G_2_/M phase cells from 5.95 ± 0.98 to 11.21 ± 1.13 (Table 1). Cell cycle distribution at 6 h after IR treatment was compared with those before IR treatment. Without SF pretreatment, cells were arrested in S phase at 6 h after radiation from 32.53 ± 2.04 to 48.20 ± 0.32 (*p* < 0.01), while SF pretreatment caused more cells to accumulate in G_2_/M phase, up to 41.51 ± 1.97 (*p* < 0.01). Usually tumor cells show a higher percentage of cells in G_1_ phase but a lower percentage in S phase after exposure to IR. However, as shown in Figure 4 and Table 1, the data showed a lower percentage distribution in G_1_ phase but higher in S phase with IR (4 Gy). In previous reports, similar results could be found whereby the A549 cells were arrested in S phase 6 h after IR [17]. This might be associated with the time of harvesting A549 cells and exposure dose rate. Since SF drove more cells to leave S phase and arrest in G_2_/M phase, which is considered to be the most sensitive stage of cell cycle to IR [18,19], this cell cycle redistribution positioned more cells in the radiosensitive phase at the time of radiation, which may be a mechanism for the SF-enhanced radiosensitivity of A549 cells.

**Figure 5:**
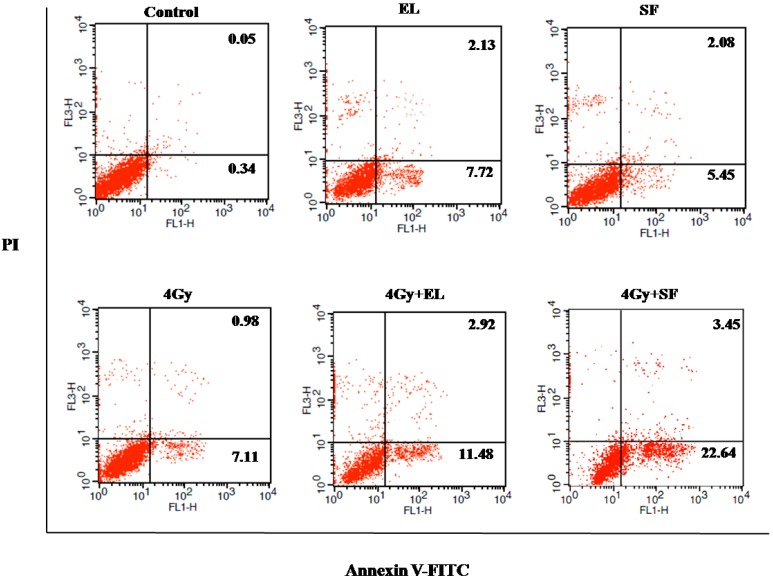
The radiosensitivity effects of β-Elemene (EL, 15 µg/mL) and schinifoline (SF, 4 µg/mL) treatment on apoptosis in A549 cells by the FACS analysis. The viable cells (Annexin V and PI negative) are in the lower left-hand quadrant. Early apoptotic cells (Annexin V positive/PI negative) are in the lower right-hand quadrant. Terminal apoptotic (Annexin V and PI positive) are in the upper right-hand quadrant.

IR-induced DNA damage followed by cell death is considered to be the mechanism for cancer cell elimination by radiotherapy. To evaluate the induction of apoptosis after the combination of SF and radiotherapy, A549 cells were treated with the 20% IC_50_ concentration of SF for 12 h before irradiation (0 or 4 Gy). Six h later, the apoptotic cells were determined by an Annexin V-FITC-PI Apoptosis Detection Kit. As the results in Figure 5 and Table 2 show, the induction of apoptosis by a low concentration of SF was similar to single exposure or EL (positive control), whereas after combination treatment, the amount of early apoptotic cells (Annexin V positive, 23.37 ± 1.75, Table 2) increased significantly and enhanced apoptosis (Annexin V positive/Annexin V and PI positive, 25.72 ± 1.31, Table 2 was observed when compared with control or IR group (*p* < 0.001), suggesting that SF rendered A549 cells more susceptible to radiation-induced apoptosis. The results were consistent with the CCK 8 test results in this paper.

**Table 1:** Percentage distribution of A549 cells.

Treatment	G_1_	S	G_2_/M
Control	61.52 ± 2.32 a	32.53 ± 2.04 a	5.95 ± 0.98 a
IR (4 Gy)	44.59 ± 0.56 b	48.20 ± 0.32 b	7.22 ± 0.37 a
SF_2_ (20% IC_50_)	58.13 ± 0.98 a	30.66 ± 0.98 a	11.21 ± 1.13 b
IR + SF_2_	32.21 ± 2.09 c	26.29 ± 1.46 c	41.51 ± 1.97 c

Means in the same column followed by the same letters do not differ significantly in ANOVA and Tukey’s tests (*p* < 0.01).

**Table 2:** The radiosensitivity effects of different treatments on apoptosis in A549 cells.

Treatment	Early Apoptotic Cells (%) ^A^	Apoptotic Cells (%) ^B^
Control	0.33 ± 0.07 a	0.39 ± 0.07 a
IR (4 Gy)	7.21 ± 0.76 b	8.06 ± 0.75 b
EL (20% IC_50_)	7.04 ± 1.54 b	8.71 ± 1.56 b
IR + EL	11.11 ± 0.63 c *	14.08 ± 0.55 c *
SF_2_ (20% IC_50_)	6.08 ± 0.94 b	7.79 ± 1.13 b
IR + SF_2_	23.37 ± 1.75 d *	25.72 ± 1.31 d *

^A^: Annexin V positive and PI negative; ^B^: Early and terminal apoptotic cells, Annexin V positive/Annexin V and PI positive; Means in the same column followed by the same letters do not differ significantly in ANOVA and Tukey’s tests (*p* < 0.05), * *p* < 0.001, *vs.* control or IR.

## 3. Experimental Section

### 3.1. General Information

^1^H- and ^13^C-NMR spectra were recorded on Bruker Avance DRX 500 NMR spectrometer with TMS as the internal standard. Silica gel (160–200 mesh, 200–300 mesh, Qingdao Marine Chemical Plant, Qingdao, China) used for column chromatography and Sephadex LH-20 were supplied by Amersham Pharmacia Biotech (Beijing, China). Analytical grade solvents were produced by Beijing Chemical Factory (Beijing, China).

### 3.2. Plant Material

Fresh pericarps (5 kg) of *Z. schinifolium* were collected at October 2012 from Kuandian Manchu Autonomous County 118200, Liaoning Province, China (40°43'46''N latitude; 124°46'41''E longitude), and identified by Dr Haibo Yin of Liaoning University of Traditional Chinese Medicine. A voucher specimen (BNU-CMH-Dushushan-2012-10-013) was deposited at College of Resources Science & Technology, Beijing Normal University. The fresh pericarps were air dried for one week and then ground to a powder.

### 3.3. Preparation of Schinifoline

The powder (3.0 kg) was extracted under ultrasound irradiation three times (each for half an hour) with methanol (10 L). The extract was concentrated under reduced pressure to obtain a crude residue (240 g). The suspension (120 g) was fractionated by silica gel column chromatography(160–200 mesh, 1000 g), using a gradient solvent system of CHCl_3_/MeOH (50:1, 30:1, 20:1, 10:1, 1:1 and MeOH) to afford ten fractions according to TLC detection on silica gel plates. Fraction 7 was re-subjected to silica gel CC and purified by chromatography on a Sephadex LH-20 column (CHCl_3_/MeOH, 1:1) to yield SF (Figure 1). Its ^1^H and ^13^C-NMR data were in agreement with the reported data [20].

### 3.4. Cell Culture and Irradiation Procedure

A549, a human lung cancer cell line, was obtained from the Chinese Academy of Medical Sciences (Beijing, China) and cultured in RPMI 1640 (Sigma, St. Louis, MO, USA) medium supplemented with 10% fetal bovine serum (GIBCO Inc, Grand Island, NY, USA), 100 IU/mL penicillin (Flow Lab, Beijing, China) and 100 μg/mL streptomycin (Flow Lab, Beijing, China) at 37 °C, 5% CO_2_.

Exponentially growing A549 cells were treated with SF prior exposure to ^60^Co γ-rays with a dose rate of 2 Gy/min in the irradiation (IR) center (Beijing Radiation Center, Beijing Academy of Science and Technology, China), and then exposed to different radiation doses to assess the optimum irradiation dose and their radiosensitivity effects.

### 3.5. Assessment of Cell Proliferative Inhibition

The viability of cells under the influence of SF was measured by a cell counting kit-8 (CCK-8) assay. The cell suspension was dispensed into a 96-well microplate at 100 μL per well (6 × 10^3^ per well). After 4–6 h preincubation in the incubator (Forma Series ΙΙ Water Jacket) to allow cellular attachment, various concentrations of test solution were added and cells were incubated for 6, 12, 24 h, respectively. At the end of the incubation, CCK-8 reagent (Cell Counting Kit-8, Dojindo, Kumamoto, Japan, 10 μL) was added into each well followed by further incubation for 2 h. The optical density (OD) was recorded at 450 nm using a microplate reader (Multiskan GO, Thermo Scientific, Waltham, MA, USA). Each determination represented the average mean of six replicates. β-Elemene (EL, Jingang Pharmaceutical Co., Dalian, China) was used as positive control [14]. The 50% inhibitory concentration (IC_50_) value was calculated the line equation of the dose-dependent curve.

A separate experiment was carried out to study the radioprotective effects of SF, A549 cells were treated with 10% (2 µg/mL) or 20% IC50 (4 µg/mL) value of SF for 12 h before exposure to different doses (0, 4, 6 or 8 Gy) of gamma radiation. Immediately after IR treatment, the media containing the chemical drugs was discarded and replaced with fresh medium. Then the cells were allowed to grow for 6, 12, 24 h, respectively until determination.

### 3.6. Colony Forming Assay

The experiment was divided into four groups: (I) control group; (II) IR group, the cells were irradiated with 0, 2, 4, 6 or 8 Gy without SF; (III) SF group, the cells were treated with 20% IC_50_ (4 µg/mL); (IV) IR+SF, the cells were exposed to 2 or 4 Gy after SF treatment. 14 days after seeding, the colonies were fixed with methanol/acetic acid (3:1) and stained with giemsa. The number of colonies containing at least 50 cells was determined, and the surviving fractions were calculated. The surviving fractions were calculated using the plating efficiency for each treatment group (IR, SF or IR + SF combination).

### 3.7. Flow Cytometric Analysis of Cell Cycle

Exponentially growing A549 cells were plated into a 6-well plate at 1 × 10^5^cells/mL and treated with SF for 12 h. Thereafter the cells were irradiated with 0 or 4 Gy and further incubated for 6 h. For the measurement of Cell Cycle Detection Kit (Key GEN BioTECH, Beijing, China) was used according to the manufacturer’s instruction. In brief, cells were collected and washed with PBS, fixed with 70% ethanol storage at 4 °C overnight. The cells were incubated with 100 μL ribonuclease A (RNase A) for 30 min at 37 °C, and then stained with 400 μL propidium iodide (PI) for 30 min at 4 °C keeping in dark place. Samples were analyzed by flow cytometry (FACSCalibur, Becton Dickinson, Shanghai, China). The percentage of cells in G_1_, S, and G_2_/M phase of cell cycle were calculated using ModFit LT software (Verity Software House, Topsham, ME, USA).

### 3.8. Flow Cytometric Analysis of Apoptosis

For the measurement of Annexin V-FITC-PI Apoptosis Detection Kit (BD Biosciences, Beijing, China) was used according to the manufacturer’s instructions. In brief, cells were collected and washed twice with PBS, suspended in 400 µL of binding buffer, stained with Annexin V-FITC and PI, followed by incubation for 15 min in the dark, and then analyzed by the flow cytometry (Becton Dickinson, FACSCalibur), counting 20,000 events per sample.

## 4. Conclusions

The present investigation suggests that schinifoline could be effective as an adjuvant in lung cancer therapy. The radiosensitization by schinifoline might be due to increased cell apoptosis and affected cell cycle distribution. Since *Z. schinifolium* is used in China for treatment of various ailments, schinifoline may offer an alternative treatment strategy for lung cancer in combination with gamma radiation. Moreover, further study is needed to investigate the internal mechanism of schinifoline in relation to radiosensitization. It is expected to be significant in human cancer chemotherapy and radiotherapy. 
